# Expression of *homothorax* and *extradenticle* mRNA in the legs of the crustacean *Parhyale hawaiensis*: evidence for a reversal of gene expression regulation in the pancrustacean lineage

**DOI:** 10.1007/s00427-008-0221-4

**Published:** 2008-05-27

**Authors:** Nikola-Michael Prpic, Maximilian J. Telford

**Affiliations:** 1 Department of Developmental Biology, GZMB New Building, Georg-August-University Goettingen, Johann-Friedrich-Blumenbach Institute for Zoology and Anthropology, Justus-von-Liebig-Weg 11, 37077 Goettingen, Germany; 2 Department of Biology, Darwin Building, University College London, Gower Street, London, WC1E 6BT UK

**Keywords:** Arthropod phylogeny, Gene expression regulation, Extradenticle, Homothorax, Appendage development

## Abstract

In *Drosophila* leg development, the *extradenticle* (*exd*) gene is expressed ubiquitously and its co-factor *homothorax* (*hth*) is restricted to the proximal leg portion. This condition is conserved in other insect species but is reversed in chelicerates and myriapods. As the region of co-expression does not differ in the two groups and transcripts from both are necessary for function, this difference in expression is likely to be functionally neutral. Here, we report the expression patterns of *exd* and *hth* in a crustacean, the amphipod shrimp *Parhyale hawaiensis*. The patterns in *P*. *hawaiensis* are similar to the insect patterns, supporting the close relationship between crustaceans and insects in the taxon Tetraconata. However, mRNA expression of *exd* in *P*. *hawaiensis* is weak in the distal leg parts, thus being intermediate between the complete lack of distal *exd* expression in chelicerates and myriapods and the strong distal *exd* expression in insects. Our data suggest that the reversal of the gene expression regulation of *hth* and *exd* occurred in the pancrustacean lineage.

## Introduction

The transcription factor *extradenticle* (*exd*) in *Drosophila melanogaster* is expressed throughout the leg. Its function, however, is required only in the proximal part of the leg (Gonzales-Crespo et al. 1998; Abu-Shaar and Mann 1998). Restriction of *exd* function to the proximal leg is achieved by spatial restriction of the gene *homothorax* (*hth*) the product of which is the co-factor of Exd (Kurant et al. 1998; Pai et al. 1998; Rieckhof et al. 1997). Both proteins are stable and nuclear only when bound to each other (Abu-Shaar et al. 1999; Berthelsen et al. 1999; Jaw et al. 2000). Ultimately, while *exd* is expressed throughout the leg, *hth* is restricted to the proximal part of the leg and stable nuclear Exd and Hth protein (and thus *exd*–*hth* function) is therefore restricted to the area of co-expression in the proximal leg part.

It is interesting to note that the reverse situation, i.e. *hth* expression throughout the leg and proximal restriction of *exd*, does not alter the area of co-expression. The two alternatives are functionally equivalent and could theoretically be realised in nature. Among insects, however, the condition seen in *D*. *melanogaster* seems to be conserved. The beetle *Tribolium castaneum* (Prpic et al. 2003), the true bug *Oncopeltus fasciatus* (Angelini and Kaufman 2004) and the cricket *Gryllus bimaculatus* (Inoue et al. 2002) also have ubiquitous *exd* expression in the leg and restricted proximal *hth* expression.

Representatives of the chelicerates and the myriapods, however, do show the reverse situation: in the spider *Cupiennius salei* (Prpic et al. 2003; Prpic and Damen 2004) and the millipede *Glomeris marginata* (Prpic and Tautz 2003) *hth* is expressed throughout most of the leg (only the distal tip lacks *hth* expression), and *exd* expression is restricted to proximal leg portions. This demonstrates that the regulation of *hth* and *exd* expression has been reversed during arthropod evolution. This reversal could have been achieved by simultaneously releasing the proximal restriction of one gene and gaining proximal restriction of the other gene in order to ensure co-expression is restricted to proximal parts at all times. Alternatively, a transitional stage would be required in which both genes are restricted to the proximal leg and subsequent release of restriction affects either one or the other gene (see also Prpic et al. 2003). In any case, the reversal of gene expression regulation of *hth* and *exd* is not a simple process which could be expected to occur frequently during arthropod evolution. Rather the reversal of *hth* and *exd* expression probably occurred only once during arthropod evolution and thus could be a useful apomorphy.

In order to locate the point in time when the reversal has taken place, representatives of all four major arthropod taxa (insects, crustaceans, myriapods and chelicerates) have to be examined for the mRNA expression of *exd* and *hth*. In the crustaceans, however, expression has only been studied using a cross-reacting antibody against Exd protein (Gonzalez-Crespo and Morata 1996; Abzhanov and Kaufman 2000; Williams et al. 2002). Unfortunately, since in the absence of Hth protein Exd protein levels are low or undetectable (Casares and Mann 1998; Pai et al. 1998; Jaw et al. 2000), Exd antibody stainings can only reliably detect the co-expression area which is expected to be identical in all arthropods. In order to discriminate between ubiquitous or proximally restricted expression of the *exd* and *hth* genes, the mRNA expression of *exd* and *hth* must be studied. The crustaceans are the only arthropod class from which data on mRNA expression of *exd* and *hth* are missing. We have therefore investigated *exd* and *hth* expression in the amphipod shrimp *Parhyale hawaiensis* in order to close this gap in the available dataset. We show that in the uniramous legs of *P*. *hawaiensis* the expression is very similar to the expression in insects. This suggests that the changes in gene expression regulation leading to the reversal of *hth* and *exd* expression have occurred before the split between crustaceans and insects and may serve as an autapomorphy of the pancrustaceans.

## Materials and methods

### *P*. *hawaiensis* culture

The animals were cultured in large plastic boxes in artificial seawater (33 g/l of synthetic sea salt). The water was constantly ventilated using membrane pumps and aquarium air stones. The animals were fed dry fish flakes twice a week and, occasionally, organic carrots. The water was changed every 2 weeks, usually after the animals had also been fed.

Embryos were collected with watchmaker forceps from gravid females that had been anaesthetised with clove oil (one drop per 50-ml seawater). Embryos were staged and fixed according to Browne et al. (2005).

### Gene cloning

Total RNA was isolated from approximately 100 embryos of different developmental stages using Trizol (Invitrogen). cDNA was transcribed with Expand Reverse Transcriptase (Roche). cDNA fragments with similarity to *hth* and *exd* from *D*. *melanogaster* were obtained by polymerase chain reaction using the primers previously published (Prpic et al. 2003). Sequence alignments and phylogenetic sequence analysis were done as described in Prpic et al. (2005). Accession numbers for the *P*. *hawaiensis* sequences are as follows: AM850852 (*Ph*-*hth*) and AM850853 (*Ph*-*exd*).

### In situ hybridisation and preparation

In situ hybridisation was performed as described in Browne et al. (2005) with minor modifications. Embryos were prepared in 80% glycerol and the appendages were dissected with sharpened tungsten wire tools. Specimens were documented with a Zeiss Axioplan compound microscope and a Zeiss digital camera. All images were corrected for brightness, contrast and colour values with Adobe Photoshop 7.0 for Apple Macintosh.

## Results and discussion

### *Homothorax* and *extradenticle* from *P*. *hawaiensis*

Data from other arthropod species show that *exd*, *hth* or both genes can be present as pairs of paralogous genes. For instance, in the spider *C*. *salei*, both genes have been duplicated (Prpic et al. 2003) and in the annotated genome sequence of the jewel wasp *Nasonia vitripennis* (Human Genome Sequencing Center 2007) two *exd* genes are present. In order to amplify from *P*. *hawaiensis* cDNA fragments with similarity to *exd* or *hth*, we used degenerate primers (see “Materials and methods”) that have previously been shown to be able to also amplify paralogous genes if present. We sequenced three clones of the *hth* assay and seven clones of the *exd* assay, and all clones contained identical sequences for each gene. Thus, we did not find evidence for paralogous genes in *P*. *hawaiensis* although the number of sequenced clones per gene is too small to confidently exclude the possibility that further paralogous genes exist.

**Fig. 1:**
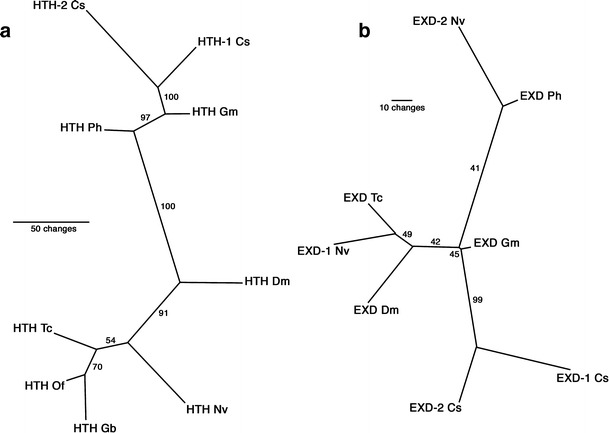
Phylogenetic analysis of selected arthropod Hth and Exd sequences. **a** Puzzle tree for homothorax sequences. **b** Puzzle tree for extradenticle sequences. All trees show the unrooted majority-rule consensus phylogram for 1,000 intermediate trees computed with the Quartet Puzzling method (Strimmer and von Haeseler 1996). Reliability values are indicated at the tree edges. Species abbreviations: *Cs*, *C*. *salei*; *Dm*, *D*. *melanogaster*; *Gb*, *G*. *bimaculatus*; *Gm*, *G*. *marginata*; *Nv*, *N*. *vitripennis*; *Of*, *O*. *fasciatus*; *Ph*, *P*. *hawaiensis*; *Tc*, *T*. *castaneum*

Next, we performed a phylogenetic analysis to establish the orthology of the sequences. The topology of the Puzzle tree for all available non-insect *hth* sequences and a selection of available insect *hth* sequences (Fig. 1a) is well supported (most edges have reliability values above 90). The *P*. *hawaiensis* sequence is grouped together with the sequences from the spider *C*. *salei* and the millipede *G*. *marginata*. The insect *hth* sequences form a separate branch. The topology of the Puzzle tree for the *exd* sequences (all available non-insect sequences and selected insect sequences; Fig. 1b) is less well supported. The *P*. *hawaiensis* sequence is joined with one of the paralogous *N*. *vitripennis* genes and there is no well-supported separation of insect and non-insect sequences. Both analyses demonstrate that the sequences from *P*. *hawaiensis* are closely related to *exd* or *hth* from other arthropods and we designate the corresponding genes as *Ph*-*exd* and *Ph*-*hth*, respectively.

**Fig. 2:**
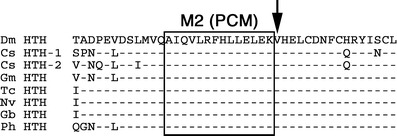
Alignment of a conserved alpha helix in Hth proteins from various arthropods. The helix is involved in Hth–Exd interactions in *D*. *melanogaster* and is highly conserved in other arthropods. The motif M2 (*boxed*) and the valine behind it (*arrow*) have been shown to be essential for protein–protein interactions (*PCM* = Pbx co-operation motif). *Dashes* indicate identical amino acid residues. Species abbreviations see Fig. 1

Studies of the *D*. *melanogaster* Homothorax protein and its vertebrate homolog, Meis, have identified two alpha helices that appear to be involved in the heterodimerization of Hth–Meis and Exd–Pbx proteins. While the data for one of these helices are ambiguous, the other helix contains a motif (M2) that has been demonstrated to bind the co-factor Exd or its vertebrate homologue Pbx (Knoepfler et al. 1997; Jaw et al. 2000). Figure 2 shows an alignment of the M2 containing helix sequences from different arthropods with the M2 motif boxed. The sequence of this motif is highly conserved in all arthropods and the valine just outside the M2 motif that has been shown to be necessary for heterodimerization (Kurant et al. 2001) is also conserved. This suggests that the heterodimerization of Hth–Meis and Exd–Pbx that is crucial for the function of these proteins in *D*. *melanogaster* and vertebrates might be conserved throughout the arthropods.

### Expression of *hth* and *exd* in *P*. *hawaiensis* leg development

We next examined the expression patterns of *Ph*-*exd* and *Ph*-*hth* in the trunk legs of *P*. *hawaiensis* by whole-mount *in situ* hybridisation. The trunk of *P*. *hawaiensis* shows the sub-tagmatization typical for amphipod crustaceans (for an overview see inset in Fig. 4). The first trunk segment is fused to the head forming a cephalothorax. The morphology of the first trunk leg pair is therefore similar to gnathal appendages (maxillae) and they are called maxillipeds. The following seven segments form the peraeon and their appendages are called peraeopods. The maxillipeds and the peraeopods are uniramous appendages. The following pleosome and urosome consist of three segments each. The appendages of these body parts—pleopods and uropods—are biramous (branched) appendages.

**Fig. 3:**
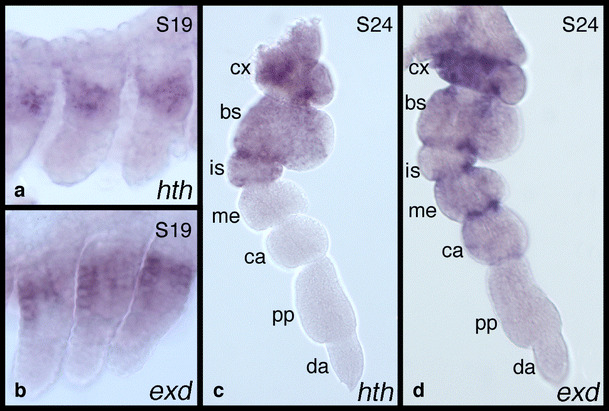
Expression of *Ph*-*hth* and *Ph*-*exd* mRNA in *P*. *hawaiensis* peraeopods. **a** Expression of *Ph*-*hth* in the peraeopods of an embryo at stage S19 (for *P*. *hawaiensis* embryonic development see Browne et al. (2005)). **b** Expression of *Ph*-*exd* in the peraeopods of an embryo at stage S19. In both cases, expression is restricted to proximal areas. **c** Expression of *Ph*-*hth* in a peraeopod at stage S24. Podomeres are indicated next to the appendage. Expression is restricted to the three proximal podomeres. **d** Expression of *Ph*-*exd* in a peraeopod at stage S24. Expression is ubiquitous but is weaker in the distal portion of the appendage. Abbreviations: *cx*, coxa; *bs*, basis; *is*, ischium; *me*, merus; *ca*, carpus; *pp*, propodus; *da*, dactylus

In early stages of peraeopod development, both genes are expressed in the proximal part of the legs (Fig. 3a,b). At later stages (Fig. 3c,d), the leg segments (podomeres) can be distinguished and the expression of *Ph*-*hth* and *Ph*-*exd* can be attributed to specific podomeres. *Ph*-*hth* shows a striking expression border between the ischium and the merus (Fig. 3c). Expression of *Ph*-*hth* is thus restricted to the proximal podomeres: coxa, basis and ischium. In contrast, expression of *Ph*-*exd* at later stages is ubiquitous in the legs but is clearly stronger in proximal podomeres (Fig. 3d). The two distal podomeres—the propodus and dactylus—express the gene at a lower level. Thus, there is a step-wise proximal-to-distal gradient of *Ph*-*exd* expression along the length of the leg.

**Fig. 4:**
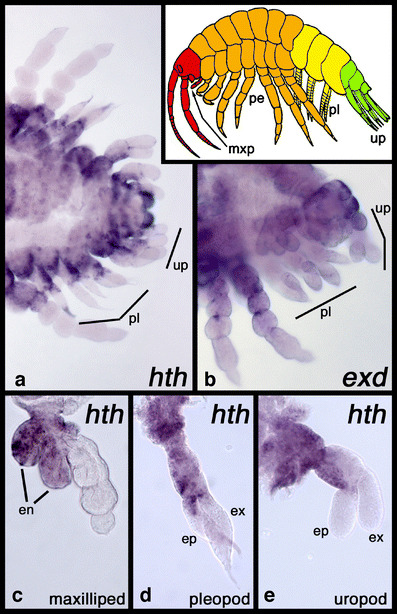
Expression of *Ph*-*hth* and *Ph*-*exd* mRNA in *P*. *hawaiensis* pleopods and uropods. **a**, **b** Rear ends of stage 24 embryos, anterior to the left. Expression of *Ph*-*hth* is excluded from distal parts of the pleopods and uropods (**a**). Please note that the dark staining at the tip of some pleopods in the figure is an artificial staining caused by attraction of the antibody towards the embryonic cuticle that starts developing at these late stages. Although the antibody was pre-absorbed against fixed old embryos, artificial staining of cuticle could not be suppressed completely in all specimens. Expression of *Ph*-*exd* is ubiquitous at a low level in the pleopods and uropods (**b**). **c**–**e** Dissected trunk legs stained for *Ph*-*hth* mRNA. **c** Maxilliped. **d** Pleopod. **e** Uropod. The *inset* shows a generalised sketch of an amphipod shrimp with the tagmata denoted with colours: *red*, cephalothorax; *orange*, peraeon; *yellow*, pleosome; *green*, urosome. Abbreviations: *en*, endites; *ep*, endopod; *ex*, exopod; *mxp*, maxilliped; *pe*, peraeopods; *pl*, pleopods; *up*, uropods

In the pleopods and uropods, *Ph*-*hth* expression is again excluded from distal parts (Fig. 4a), whereas *Ph*-*exd* is ubiquitously expressed in these legs at a level comparable to the level of *Ph*-*exd* expression in the two distal podomeres of the peraeopods (Fig. 4b). However, the spatial expression of *Ph*-*hth* in the pleopods and uropods is not simply restricted to the proximal leg parts and is thus not directly comparable to the expression in the peraeopods. In the pleopods, the inner branch (endopod) expresses *Ph*-*hth* in its proximal third (Fig. 4d) and in the uropods only the ventral half of the basis expresses *Ph*-*hth*, thus leading to a striking border between the dorsal and the ventral portion of this podomere. By contrast, the expression in the maxillipeds is very similar to the expression in the peraeopods. In this appendage, *Ph-hth* is restricted to the proximal podomeres and the two endites growing from them on the ventral side.

### *Ph*-*hth* marks serially homologous areas in uniramous but not biramous legs

In the case of *Ph*-*hth*, the expression in the serially homologous leg types does not seem to mark serially homologous portions in all of them. Rather, the expression of *Ph*-*hth* in the pleopods and uropods is more reminiscent of a cell lineage marker. In the uropods in particular, the expression in the basis indicates the presence of a dorsal–ventral clonal border (compartment border) within this leg segment. Thus, the expression data suggest that *Ph*-*hth* expression in the different trunk leg types does not in all cases indicate homologous portions, but rather the initial population of *Ph*-*hth*-expressing cells may have different numbers of offspring in the various leg types resulting in different expression patterns. An exception appears to be the two uniramous leg types because the patterns in maxilliped and peraeopod are very similar and it is likely that the proximal portions in these legs expressing *Ph*-*hth* are indeed serially homologous. More data also from other proximally expressed genes are necessary to corroborate this issue.

### A reversal of gene expression regulation in the pancrustacean lineage

In all arthropods studied so far, co-expression of *hth* and *exd* mRNA is restricted to the proximal part of the leg and the Exd–Hth interaction motif M2 is also identical in all species. This supports a role of *exd*–*hth* co-operation in proximal leg specification in all arthropods. The gene expression patterns of both genes in *P*. *hawaiensis*, however, are clearly different from the patterns observed in chelicerates and myriapods and are more similar to the insect patterns. This finding is consistent with a close relationship between crustaceans and insects in the taxon Tetraconata that excludes the myriapods.

**Fig. 5:**
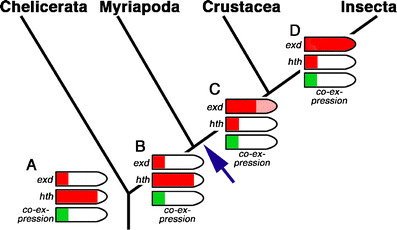
Hypothesis for the transition from *exd*–*hth* expression in chelicerates to *exd*–*hth* expression in insects. The *cartoons* represent the ancestral states (except for *D* which represents an apomorphic state) for the respective group and are deduced from the extant patterns. The *red cartoon legs* show the expression of *exd* (*top*) and *hth* (*bottom*). The *green cartoon legs* show the area of co-expression. Note that this area is identical in all cases and denotes the area of Exd–Hth function. *A* is hypothesized to be the ancestral condition in the arthropods and is retained in the extant Chelicerata and is also unchanged in the common ancestor of Myriapoda, Crustacea and Insecta (*B*) and the extant Myriapoda. *C* represents the ancestral state for the Crustacea and Insecta and is retained in the Crustacea, whereas in the insect lineage a new apomorphic state is evolved (*D*). *A* is based on Prpic et al. (2003) and Prpic and Damen (2004). *B* is based on Prpic and Tautz (2003). *C* is based on this paper. *D* is based on Angelini and Kaufman (2004), Inoue et al. (2002) and Prpic et al. (2003). The *blue arrow* denotes the origin of the gene expression regulation reversal by the release of *exd* proximal restriction and the proximal restriction of *hth* expression. In this view, the expression patterns in crustaceans mediate between the reversed conditions in chelicerates–myriapods and insects. For details, please see text

It is not currently possible to use the changes in *hth* and *exd* expression regulation directly as apomorphies for cladistic purposes because the plesiomorphic state is unknown. This would require studies of mRNA expression of these genes in arthropod outgroups like onychophorans or tardigrades. However, by mapping the expression data on a phylogenetic tree derived from other data, the likely ancestral state can be inferred and evolution of expression regulation can be reconstructed. For this approach, we use the best currently supported phylogenetic tree (see Fig. 5). The phylogenetic position of the myriapods is debated, but most data suggest a sister group relationship with the crustacean–insect clade (i.e. the Mandibulata hypothesis; Harzsch et al. 2004; Giribet et al. 2001).

Based on this tree, the chelicerates are the most basal branch and their *exd*–*hth* pattern represents the ancestral state for the arthropods (Fig. 5, A). The pattern remains virtually unchanged in the myriapods (Fig. 5, B). However, in the pancrustacean (= tetraconatan) lineage, proximal restriction of *exd* expression has been released (Fig. 5, blue arrow). As we have shown here, the expression border of *exd* in crustaceans is shifted distally and a low amount of *exd* mRNA is detected even in the distalmost podomeres. In combination with an almost ubiquitous expression of the *hth* partner, this would lead to a proximalisation of the entire leg. In order to avoid this, the regulation of *hth* expression has also been changed to restrict *hth* to the proximal part of the legs (Fig. 5, C). Finally, in the insects, *exd* expression has achieved high levels in distal areas and *hth* expression remains restricted to the proximal parts, thus leading to a reversal of expression patterns as compared to chelicerates and myriapods (Fig. 5, D). In this way, the expression patterns in crustaceans would be intermediate between the two extremes in chelicerates–myriapods on the one hand and insects on the other hand. Note that *exd*–*hth* co-expression and thus stable nuclear Exd and Hth protein in all cases is identical (see green leg cartoons in Fig. 5). The different patterns in the four arthropod groups are thus theoretically functionally fully equivalent.

Our hypothesis rests on two assumptions that need to be tested further. First, it is necessary to study more species from each arthropod class in order to clarify the variability of *exd* and *hth* expression within different arthropod groups. For the time being, our idea of *exd* and *hth* expression in three out of four arthropod classes is based on a single species only. Second, our scenario is based on an arthropod phylogeny that assumes that chelicerates are basal arthropods and that Mandibulata is a monophyletic group. For instance, if the alternative arthropod phylogeny that joins myriapods and chelicerates (Paradoxopoda or Myriochelata concept) is considered (see Harzsch et al. (2004) and Giribet et al. (2001) for a discussion of this concept), the similar expression patterns in myriapods and chelicerates could be interpreted as a synapomorphy of these taxa. In this context, it would also be important to study outgroups to determine the ancestral state of *exd* and *hth* expression in the uniramous legs.
